# The Notch ligand DLL1 exerts carcinogenic features in human breast cancer cells

**DOI:** 10.1371/journal.pone.0217002

**Published:** 2019-05-20

**Authors:** Joana Sales-Dias, Gabriela Silva, Márcia Lamy, Andreia Ferreira, Ana Barbas

**Affiliations:** 1 iBET—Instituto de Biologia Experimental e Tecnológica, Oeiras, Portugal; 2 ITQB—Instituto de Tecnologia Química e Biológica, Oeiras, Portugal; 3 Bayer Portugal, Carnaxide, Portugal; University of South Alabama Mitchell Cancer Institute, UNITED STATES

## Abstract

Breast cancer (BC) is the most common type of cancer in women and has a high rate of relapse and death. Notch signaling is crucial for normal breast development and homeostasis. Dysregulation of Notch receptors and ligands has been detected in different BC subtypes and shown to be implicated in tumor development, progression, drug resistance, and recurrence. However, the effects of Notch ligands in various types of BC remain poorly understood. In this study, we investigated the effects of the Notch ligand DLL1 in three different human BC cell lines: MCF-7, BT474, and MDA-MB-231. We showed that DLL1 expression is higher in MCF-7 and BT474 than in MDA-MB-231 cells, and that these cells respond differently to DLL1 downregulation. Functional assays in MCF-7 cells demonstrated that siRNA-mediated DLL1 downregulation reduced colony formation efficiency, migration, proliferation, caused cell cycle arrest at the G1 phase, and induced apoptosis. Gene expression studies revealed that these effects in MCF-7 cells were associated with increased expression of the cell cycle arrest p21 gene and decreased expression of genes that promote cell cycle progression (CDK2, SKP2), and survival (BCL2, BIRC5), unravelling possible mechanisms whereby DLL1 downregulation exerts some of its effects. Moreover, our results demonstrate that treatment with recombinant DLL1 increased MCF-7 cell proliferation and migration, confirming that DLL1 contributes to these processes in this BC cell line. DLL1 downregulation reduced the colony formation efficiency of BT474 cells and decreased the migration and invasion abilities of MDA-MB-231 cells but showed no effects in the proliferation and survival of these cells.

**Conclusions:**

These findings provide further evidence that DLL1 exerts carcinogenic effects in BC cells. The dissimilar effects of DLL1 downregulation observed amongst MCF-7, BT474, and MDA-MB-231 cells is likely due to their distinctive genetic and biologic characteristics, suggesting that DLL1 contributes to BC through various mechanisms.

## Introduction

Breast cancer is the most common cancer in women worldwide, and besides being the second leading cause of death by this malignancy, it also accounts for nearly 30% of new cancer diagnosis [1]. BC is a highly heterogeneous disease that can be classified into various types based on pathology, tumor grade and stage, and gene expression profile. According to the gene expression signature BC can be divided into 4 subtypes: luminal A and luminal B (positive for the oestrogen and progesterone receptors (ER^+^ and PR^+^)), HER2^+^ (human epidermal growth factor receptor), and triple-negative breast cancers (TNBC) [2]. The luminal A tumors (ER^+^, PR^+^, HER2^-^), which represent the most common BC subtype, have high expression of ER-related genes and lower expression of proliferative genes when compared to luminal B cancers (ER^+^, PR^+^, HER2^+)^. Luminal B tumors tend to be of higher grade than luminal A and their prognosis is slightly worse. Triple-negative breast cancers include a heterogeneous subgroup of tumors that lack expression of the ER and PR hormone receptors, as well as of the HER2 protein, and exhibits the most aggressive phenotype and a poor clinical outcome [2]. Despite early detection and targeted therapy, tumor recurrence and metastasis are the main cause of death in BC patients [1]. Understanding the mechanisms implicated in BC is therefore crucial for the design of more effective and targeted therapies.

The Notch signaling pathway is an evolutionarily conserved cell-to-cell communication system composed of four receptors (NOTCH1-4) and five ligands (JAG1, JAG2, DLL1, DLL3 and DLL4) crucial for embryonic development and tissue homeostasis [3]. Binding of the extracellular region of a membrane-bound Notch ligand in one cell to a Notch transmembrane receptor on a neighboring cell triggers Notch pathway activation, which results in the transcription of numerous Notch-target genes that regulate various cellular processes, including maintenance and self-renewal of stem cells, cell fate determination, growth, and survival. The diversity of functional outcomes of Notch signaling is dependent on many different regulatory mechanisms, such as receptor/ligand post-translational modifications, nuclear landscape, and crosstalk with other signaling pathways [4,5].

The Notch pathway plays an important role in normal breast biology and it has been reported to be a key oncogenic pathway in BC [5–7]. Its aberrant activation by virtue of mutations or overexpression of its receptors and/or ligands has been detected in BC, correlated with tumor initiation and progression, and more aggressive BC forms [4,6,8,9]. Notch receptors and its ligands JAG1, JAG2 and DLL4 are highly expressed in human breast carcinomas. NOTCH1 and JAG1 levels correlate with poor prognosis and poor patient overall survival [4,10,11]. NOTCH4 is associated with BC stem cell activity and drug resistance [12], JAG2 is related to the invasiveness and survival of cancer cells [13], and DLL4 overexpression is linked to tumor metastasis [14].

DLL1 expression was shown to be undetectable in normal breast tissues but moderate to high in BC [8], therefore being implicated in this pathology. Work from Chakrabarti and co-workers showed that DLL1 is highly upregulated in ER^+^ BC when compared to both normal breast tissue and TNBC tumors. In addition, DLL1 overexpression correlates with poor survival of ER^+^ BC patients, and promotes growth and metastasis of ER^+^ tumors by promoting cell proliferation, maintenance of BC stem cells and angiogenesis [15]. Another study showed that siRNA-mediated downregulation of DLL1 in BC cells inhibited their migration and invasion abilities [16]. Despite these advances in unravelling DLL1 role in BC, a better understanding of its function in this very complex disease is critical for development of more effective therapies.

In this study, we examined the role of DLL1 in luminal A (MCF-7), luminal B (BT474) and triple-negative, claudin-low (MDA-MB-231) BC cell lines and showed that DLL1 contributes to their carcinogenic features. In MCF-7 cells, siRNA-mediated DLL1 downregulation decreased colony formation, migration, cell proliferation, arrested cells in the G1 phase, and induced apoptosis. On the other hand, treatment of MCF-7 cells with recombinant DLL1 protein, which induces Notch pathway activation, lead to an increase in cell migration and proliferation. In BT474 cells DLL1 downregulation impaired colony formation efficiency while in MDA-MB-231 cells it reduced their migration and invasion abilities. Consistent with its functional effects in MCF-7 cells, DLL1 downregulation lead to a decreased expression of genes that promote cell cycle progression, proliferation and survival, and increased the levels of p21, thereby unravelling possible mechanisms underlying the carcinogenic effects of DLL1 in MCF-7 cells.

## Material and methods

### Cell culture and reagents

MCF-7 (ATCC HTB-22) and MDA-MB-231 (ATCC HTB-26), human breast adenocarcinoma cell lines, were cultured in high glucose, pyruvate DMEM medium (#41966) supplemented with 10% heat-inactivated fetal bovine serum (FBS), 100 μg/ml penicillin and streptomycin, and 0.1 mM non-essential amino acids in the case of MCF-7 cells. BT474 (ATCC HTB-20), human ductal carcinoma cells, were cultured in RPMI 1640 medium (#61870) containing 10% FBS, 100 μg/ml penicillin and streptomycin. Cells were incubated at 37°C in a humidified atmosphere and 5% CO_2_. Culture media was replaced every 2–3 days. HEK293E6 suspension cells [17] were cultured in FreeStyle F17 media (#A13835) supplemented with 4 mM glutamax, 1% pluronic F-68 and 25 mg/mL geneticin, in a 93% air/7% CO2 atmosphere at 37°C and 110 rpm agitation. For protein production cell culture media without geneticin was used. Media and cell culture reagents were from Gibco (Grand Island, New York, USA). The Notch pathway signaling inhibitor DAPT (Sigma, D5942) was used at 5 μM.

### Construction of recombinant human DLL1 expression plasmid, protein production, and purification

The cDNA fragment containing the human DLL1 signal peptide and sequences encoding the full extracellular domain was amplified by polymerase chain reaction from human DLL1 cDNA (Sino Biological, #HG11635-M, Beijing, China) using the primers: Fw: 5´- ATAGAATTCGCCGCCACCATGGGCAGTCGGTGCGCGCTGGC-3´; and Rev: 5´- GTCAGATCTGGATCCACGCGGAACCAGCCAGGGGAATGGCCCGCCCTG-3´. The amplified cDNA was cloned into the mammalian pFUSE-IgG1-Fc vector (InvivoGen, pfuse-hg1fc1, Toulouse, France) in frame with Fc tag at the C-terminal region, using standard techniques. The resulting DLL1 expression plasmid (pFUSE-DLL1-Fc) was confirmed by restriction and sequencing analyses. For the production of DLL1-Fc and Fc recombinant proteins, exponentially growing HEK2932E6 cells were transfected with the generated pFUSE-DLL1-Fc and pFUSE-IgG1-Fc vectors (1μg DNA/1.5–1.8 x10^6^ cells/mL) using polyethylenimine (Polysciences, #23966–2, Eppelheim, Germany) as transfection reagent (DNA:polyethylenimine ratio of 1:2). Cells were harvested 6–7 days post transfection. For the purification of DLL1-Fc protein, the cell culture bulk was clarified by centrifugation, and the protein present in the resulting supernatant purified by Protein-A affinity and size exclusion (SEC) chromatography on Äkta purification systems. For the purification of control Fc protein, cells expressing Fc were disrupted by homogenization (APV homogenizer 2000) before the centrifugation step. Protein purity was evaluated by SDS-PAGE and analytical SEC and concentration determined by the Bradford method.

### siRNA transfection

MCF-7 and MDA-MB-231 cells, seeded at 3–4 x10^4^ cells/cm^2^, were transfected 24 hours after seeding at 60% confluency with 100 nM of Dharmacon Accel DLL1-siRNAs (A-013302-15 and A-013302-16 herein referred as DLL1-siRNA1 and DLL1-siRNA2, respectively, Colorado, USA) and control non-targeting siRNA (D-001910-01) using HiPerFect transfection reagent (Qiagen, La Jolla, California, USA) as per manufacturer instructions. BT474 cells were transfected shortly after seeding (4–8 x10^4^ cells/cm^2^) as above. As a control for the transfection efficiency, cells were transfected with fluorescent-labelled control non-targeting siRNA (D-001950-01). After 24 hours, transfection efficiency was monitored and medium replaced by fresh culture media.

### RNA purification and quantitative real-time RT-PCR (qRT-PCR)

This was performed as previously described [18]. Briefly, total RNA was obtained using RNeasy Mini kit performing an on-column digestion of DNA with the RNase-free DNAse Set (both from Qiagen, La Jolla, CA) following the manufacturer's instructions. cDNA was generated from equal amounts of RNA by reverse transcription using the Advantage RT-for-PCR kit (Clontech Laboratories, Mountain View, CA) as per the manufacturer's instructions. Amplifications were carried out using the Roche LightCycler 480 using specific primers and FastStart DNA SYBR Green I mix (Roche Applied Science, Penzberg, Germany). All experiments were performed in triplicate or quadruplicate as per the manufacturer's instructions. mRNA transcripts were normalized to hypoxanthine-guanine-phosphoribosyltransferase (HPRT1) levels in the same sample, and the results were calculated using the advanced relative quantification method. The relative mRNA expression levels were calculated as fold change versus control samples. The expression levels of cell cycle genes were first evaluated in cDNA samples obtained from RNAs of two independent assays with the RT^2^ First Strand Kit by using the human cell cycle PCR Array PAHS-020ZG and the RT^2^ SYBR Green qPCR Mastermix (all from Qiagen) as per supplier´s instructions. Data analysis of cell cycle PCR Array was performed using the web-based data analysis software from Qiagen, and the results were then confirmed using qRT-PCR. The primers used in these assays are listed in Table 1.

**Table 1:** Primers used in gene expression analysis.

Primers	Forward (5´- 3´)	Reverse (5´- 3´)
BCCIP	TCAAGAGTTGGTTCTACGCTTC	CATGGGCAGAGCGATCTGT
BCL2	ATGTGTGTGGAGAGCGTCAACC	TGAGCAGAGTCTTCAGAGACAGCC
BIRC5	GTTGCGCTTTCCTTTCTGTC	TCCGCAGTTTCCTCAAATTC
CDC25A	TGACATCTTTCAGCTCATCG	CAGACAAAGTGGCTGTCACAG
CDK2	ATGGAGAACTTCCAAAAGGTGGA	CAGGCGGATTTTCTTAAGCG
Cyclin D2	GCGGGATATCGACCTGTGA	ACCAGATTATGGACGCGTCTCT
DLL1	CTTCCCCTTCGGCTTCAC	GGGTTTTCTGTTGCGAGGT
HEY1	GTTCGGCTCTAGGTTCCATGT	CGTCGGCGCTTCTCAATTATT
HEYL	GGAAGAAACGCAGAGGGATCA	CAAGCGTCGCAATTCAGAAA
HPRT1	CCTGGCGTCGTGATTAGTGAT	AGACGTTCAGTCCTGTCCATAA
MAD2L1	CGGACTCACCTTGCTTGTAAC	TCCAGGACCTCACCACTTTC
p15	CGTGGGAAAGAAGGGAAGAGT	GCGGCCCGGATAATCC
p21	GAGACTCTCAGGGTCGAAAACG	ATTAGGGCTTCCTCTTGGAGAAG
PCNA	GGCGTGAACCTCACCAGTAT	TCACTCCGTCTTTTGCACAG
SKP2	AGCCCGACAGTGAGAACATC	GAAGGGAGTCCCATGAAACA

### Immunoblotting analysis

Cells were lysed in Ripa buffer (Cell Signaling, #9808), containing protease inhibitors (Roche, #04693132001). Protein concentration in total soluble protein extracts was determined by using the Pierce coomassie plus assay reagent (Thermo scientific, #23238, Bleiswijk, Netherlands) according to the manufacturer’s instructions. 20–50 μg proteins were resolved by 4–12% SDS-PAGE, transferred to polyvinylidene fluoride membranes using a Bio-Rad wet system (18 hours, 4°C), and blocked (5% skimmed milk in Tris-buffered saline containing 0.1% Tween 20) for 1 hour at room temperature. Membranes were immunoblotted (18 hours, 4°C) with antibodies directed against BCL2 (#15071), BIRC5/Survivin (#2808), CDK2 (#2546), p21 (#2947), SKP2 (#2652), all from Cell Signalling, DLL1 (Abcam ab84620), and α-tubulin (Sigma, T6199). Primary antibodies were detected using anti-goat (Sigma, A5420), anti-mouse (Sigma, A9309), or anti-rabbit (Sigma, A9169) HRP-conjugated IgGs. Blots were developed with the enhanced chemiluminescence substrate western lightning plus ECL reagent (PerkinElmer, Groningen, The Netherlands) and digital images acquired in a Bio-Rad imaging system. Protein band intensities were quantified by densitometry analysis using the ImageJ Software. The relative expression levels were calculated as fold change *versus* control samples, after normalization to α-tubulin band intensities in the same sample.

### Colony formation assay

To assess colony formation, MCF-7 and MDA-MB-231 cells were collected 24 hours after transfection, (i.e. 48h post-seeding), washed once with PBS and seeded in 12-well plates at a density of 250, 500, 750, and 1000 cells/well in complete media and cultured for 7–9 days. BT474 cells were collected 48 hours after transfection/seeding and 5000–10000 cells were seeded in 6-well plates. The medium was replaced every 3 to 4 days and the cells were monitored by microscopy for colony formation at various days. At day 7–9, when well-developed colonies were detected under the microscope, the cells were fixed with ice-cold 100% methanol for 15 minutes and stained with 0.5% crystal violet in PBS/20% methanol for 15 minutes. Cell images were acquired using a Leica DMI6000 inverted microscope at a magnification of 50x. The number of colonies was determined by counting at least 6 representative fields of view from triplicate wells for each cell line and condition tested.

### Scratch wound-healing assay

Fifty-five to seventy hours after transfection, MCF-7 and MDA-MB-231 cells transfected with siRNAs and control non-transfected cells at 90% confluency were submitted to serum starvation for 16 hours in media with 0.5% FBS and scratches (2 lines across each well/condition) were created thereafter by scrapping with a 10 μL pipette tip. To evaluate the effect of DLL1-Fc protein in MCF-7 cells they were plated at 3–4 x10^4^ cells/cm^2^ on 24-well plates not coated (control) or pre-coated with DLL1-Fc or Fc proteins (2.5 μg/mL PBS, 500 μL/well, 4°C for 17 hours followed by 1 hour blocking with 1% BSA). Fifty-six hours after seeding, cells at 80–90% confluency were serum-starved, and scratches were performed as above. The scratched monolayers were then washed twice with serum free media and incubated in complete media. Images of 3 different areas of the wound of each scratch were taken with a Leica DMI6000 microscope (50x magnification) at 0 hours and 20–72 hours after scratches. The area of each wound was quantified using ImageJ software and the % of wound closure calculated as follows: [1-(wound area at t = 24–72 h/wound area at t = 0 h)×100].

### Transwell migration and invasion assays

The migratory and invasive capabilities of the cells were examined in 24-well transwell chambers with 8.0-μm pore size polyethylene terephthalate membranes, non-coated or coated with matrigel (Corning, #354578 and #354480, Massachusetts, USA), respectively. To evaluate the effect of DLL1 downregulation in cell migration and invasion potentials, non-transfected and siRNA-transfected cells were collected 48–72 hours post transfection and plated at a density of 1–2 × 10^5^ (MCF-7 and BT474) or 5 × 10^4^ (MDA-MB-231) cells in the inserts in 100 μL media with 0.5% FBS. To assess the effect of DLL1-Fc protein in the migratory capability of MCF-7, 4 × 10^4^ cells/cm^2^ were plated on 24-well culture plates not coated (control) or pre-coated with DLL1-Fc or Fc proteins (2.5 μg/mL PBS, 500 μL/well, 4°C for 17 hours followed by 1 hour blocking with 1% BSA). After 72 hours, cells were collected and 1 × 10^5^ cells were plated in the inserts of non-coated chambers as above. In both assays, bottom chambers were filled with 600 μL media supplemented with 10% serum. The cells were incubated for 14–24 hours (MDA-MB-231) or 48–72 hours (MCF-7 and BT474). Thereafter, the non-invading or non-migrating cells were removed from the top chamber with cotton swabs and PBS. Invading and migrating cells on the bottom of the membrane were fixed in 100% ice-cold methanol for 15 minutes and stained with 0.5% crystal violet in 20% methanol for 15 minutes. Invasion and Migration were quantitated by counting cells in 15 randomly-selected fields using a Leica DMIRB microscope at a magnification of 200x.

### Cell growth

Cell growth was determined 48, 72, 96, and 120 hours following transfection with siRNAs or treatment with recombinant proteins by microscopy using the trypan blue exclusion method and the MTT (Sigma, M2120) colorimetric method. For the MTT assays, cells were plated in 96-well plates and transfected as indicated above. At the end of the experiment, cells were incubated in 120 μl fresh media containing 20 μl of MTT solution (5 mg/ml). After 2–4 hours, the resulting MTT crystals were dissolved in DMSO (120 μl/well) and absorbance values were determined at 570 nm using a microplate reader. Each condition was tested in triplicate or quadruplicate for all assays.

### Cell cycle and apoptosis assays by flow cytometry

Cell cycle progression was determined by using propidium iodide (PI). Cells were harvested at 72, 86, and 98 hours post-transfection, washed with PBS (300 g, 6 minutes, 4°C) and fixed with ice-cold 70% ethanol (4°C for 4–16 hours). Thereafter, cells were washed three times in PBS and resuspended in the FxCycle PI/RNA staining solution (Thermo Fisher, #F10797, Oregon, USA) for 30 minutes at 37°C. Samples were analyzed on a Guava easyCyte flow cytometer (Millipore). Results were calculated using the cell cycle program from the FlowJo software. For the analysis of apoptosis, siRNA transfected, and control non-transfected cells were collected at 72, 86, and 120 hours post-transfection and stained with FITC-conjugated annexin-V (Biolegend, #640906, California, USA) according to the manufacturer’s instructions. Data was analyzed using the FlowJo software (Tree Star, Inc.).

### Statistical analysis

All results are presented as mean + standard deviation (SD) of at least three independent experiments. Student´s t-test was used to evaluate the significance of differences between two groups, and one-way ANOVA to evaluate differences amongst more than two groups. *P* values < 0.05 were considered statistically significant.

## Results

### DLL1 downregulation decreases the colony formation capability of luminal MCF-7 and BT474 BC cells

**Fig 1:**
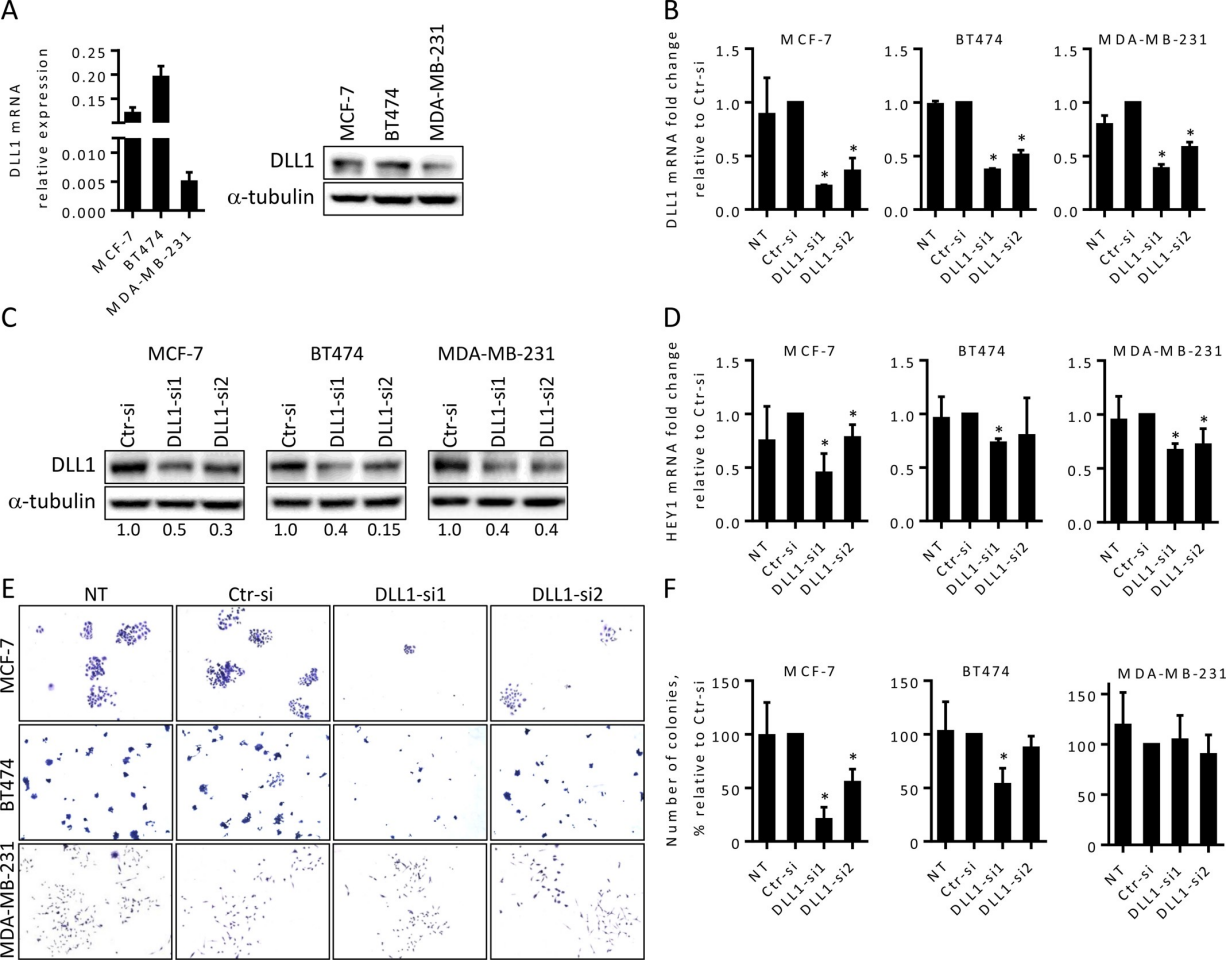
DLL1 downregulation impairs Notch pathway activation in BC cells and decreases MCF-7 and BT474 colony formation abilities. (A) Expression of DLL1 mRNA and protein levels in luminal A MCF-7, luminal B BT474, and triple-negative MDA-MB-231 cells were quantitated by qRT-PCR and immunoblotting. The DLL1 mRNA values were normalized against the HPRT1 mRNA levels in the same samples. α-tubulin was used as the loading control. (B-F) Cells were transiently transfected with DLL1 siRNAs (DLL1-si1/2), negative control siRNA (Ctr-si) or not-transfected (NT). (B) Total RNA was extracted from the indicated cells 24–38 hours post-transfection and DLL1 mRNA levels were quantitated by qRT-PCR. (C) Total soluble protein extracts were prepared from cells 72 hours following transfection and the levels of DLL1 protein were assessed by immunoblotting. The numbers under the bands indicate DLL1 fold changes relative to Ctr-siRNA transfected cells after normalization against α-tubulin. (D) qRT-PCR analysis of HEY1 mRNA in the indicated cells at 48–72 hours after transfection. The values in (B) and (D) were normalized against the HPRT1 mRNA levels in the same sample and calculated as mean fold change (+ SD) relative to the respective control cells transfected with Ctr-siRNA. Graphs in (A, B and D) represents mean (+ SD) of at least three independent assays. (E-F) Colony formation of MCF-7, BT474 and MDA-MB-231 cells at day 9 post transfection. Representative fields of crystal-violet-stained colonies for each cell type of four independent assays are shown. The graphs show mean percentage of the number of colonies (+ SD) relative to the respective control cells transfected with Ctr-siRNA from these assays. *, *P* < 0.05, compared with Ctr-siRNA transfected cells at the respective experimental condition.

To assess the relevance of DLL1 in BC, we first examined the expression levels of DLL1 in three different tumorigenic BC cell lines: poorly aggressive luminal A MCF-7 and luminal B BT474 cells, that express the estrogen receptor and retain an epithelial morphology, and the highly aggressive, invasive and poorly differentiated TNBC MDA-MB-231 cells [19]. Our qRT-PCR and immunoblotting results revealed that all three cell lines express DLL1, with MCF-7 and BT474 cells presenting higher levels than MDA-MB-231 cells (Fig 1A).

To investigate the functional role of DLL1 in these BC cells, MCF-7, BT474, and MDA-MB-231 cells were transfected with two DLL1 specific siRNAs (DLL1-siRNA1 and DLL1-siRNA2), to downregulate DLL1 expression, and with a non-targeting siRNA (Ctr), as a negative control. siRNA-mediated DLL1 downregulation was confirmed by qRT-PCR and immunoblotting. Our results showed that DLL1 mRNA levels were decreased by 80% in MCF-7 cells and by 60% in BT474 and MDA-MB-231 cells, upon transfection with DLL1-siRNA1, in comparison to cells transfected with Ctr-siRNA (Fig 1B). Transfection with DLL1-siRNA2 also led to a significant reduction of DLL1 mRNA levels in the cell lines under study but the reductions were smaller than those obtained with DLL1-siRNA1 (Fig 1B). Analysis of the protein levels showed that DLL1 was decreased in all the cell lines transfected with the specific DLL1-siRNAs. Consistent with the mRNA data, transfection with DLL1-siRNA1 led to a significantly higher reduction in the DLL1 protein levels in MCF-7 and BT474 cells, when compared to DLL1–siRNA2 (Fig 1C).

As DLL1 exerts its biological effects via activation of the Notch signaling pathway we next evaluated the expression levels of the HEY1 gene, a classical Notch-target gene [4], upon DLL1 downregulation as a functional readout of DLL1-Notch pathway activation. Our results showed that DLL1 downregulation by DLL1-siRNA1 was associated with an impairment in the activation of the Notch signaling pathway in all cell lines, as evidenced by the significant reduction in the expression levels of HEY1 (Fig 1D). qRT-PCR analysis revealed that the HEY1 levels were approximately 50% lower in MCF-7 cells and 30% reduced in BT474 and MDA-MB-231 cells transfected with DLL1 siRNA1, when compared to the respective controls. Consistent with a lower downregulation of DLL1 mRNA expression, HEY1 expression levels were only 30% diminished in MCF-7 cells transfected with DLL1-siRNA2. Transfection with DLL1-siRNA2 also inhibited HEY1 levels by nearly 30% in MDA-MB-231 cells, in comparison to control cells. However, transfection of BT474 cells with DLL1-siRNA2 showed this siRNA did not significantly affected the expression levels of HEY1 (Fig 1D). These results show that transfection with DLL1-siRNA1 results in a higher downregulation of DLL1 expression and DLL1-mediated Notch pathway signaling activation in MCF-7 and BT474 cells. Since DLL1 has been shown to promote clonogenic growth of cancer cells *in vitro* [20], we assessed whether its downregulation could impact the colony formation capability of BC cells. To test this, cells transfected with the specific DLL1-siRNAs and Ctr-siRNA were collected 24–48 hours post-transfection, seeded at very low densities, and colony formation was monitored by microscopy at various days. Non-transfected cells were used as control for siRNA transfections. A decrease in the number of colonies of MCF-7 and BT474 cells transfected with DLL1 siRNAs was observed starting at day 4 after seeding at low densities. Analysis of the number of colonies at day 7–9, when the colonies were larger, showed that DLL1 downregulation by DLL1-siRNA1 lead to a decrease in the number of MCF-7 and BT474 colonies by 80% and 50%, respectively, when compared to the corresponding controls (Fig 1E and 1F). DLL1 downregulation by DLL1-siRNA2 caused a 45% reduction in the number of MCF-7 colonies formed, compared to Ctr-siRNA. These results are in agreement with the observations that DLL1-siRNA1 had a higher impact in the DLL1 levels and Notch pathway activation in comparison to DLL1-siRNA2 (panel 1 in Fig 1B–1D, respectively). In BT474 cells DLL1-siRNA2 did not cause a significant decrease in colony forming ability, which is in agreement with our data showing that this siRNA did not impair the activation of the Notch pathway in a consistent manner (Fig 1D–1F). Contrary to MCF-7 and BT474 cells, no significant changes were observed in the colony formation ability of MDA-MB-231 cells upon DLL1 downregulation with both siRNAs (Fig 1E and 1F). These results indicate that DLL1 is required for colony formation in BC luminal MCF-7 and BT474 cells.

Considering the results described above showing higher DLL1 downregulation and effects in MCF-7 and BT474 cells upon transfection with the DLL1-siRNA1, this siRNA was the one chosen to further study the effect of DLL1 downregulation in these cells. Studies in MDA-MB-231 were performed using both DLL1 siRNAs.

### DLL1 downregulation decreases MCF-7 cell migration and MDA-MB-231 cell migration and invasion abilities

**Fig 2:**
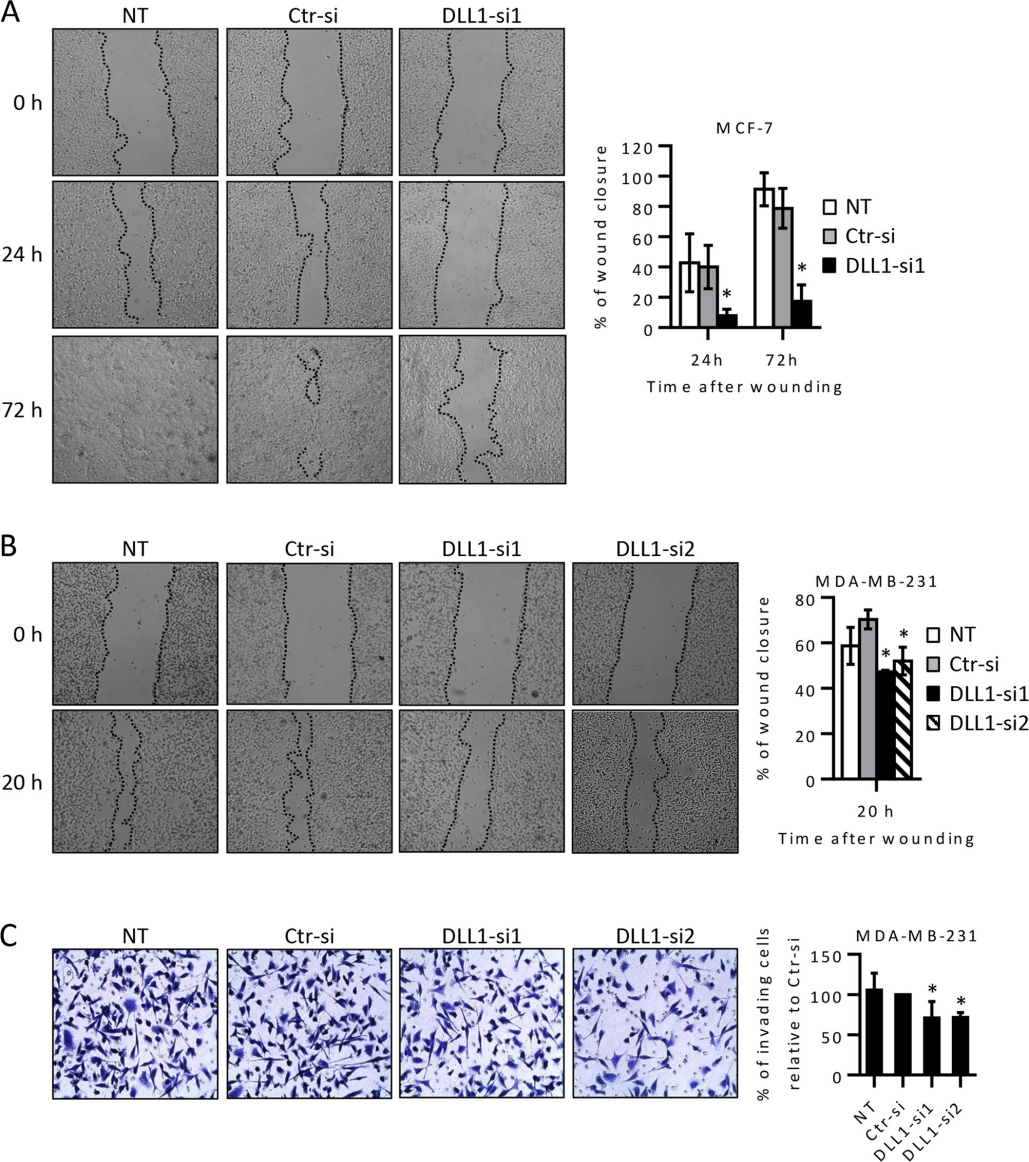
DLL1 downregulation decreases migration of MCF-7 and MDA-MB-231 cells and the invasive potential of MDA-MB-231 cells. MCF-7 and MDA-MB-231 cells were transiently transfected with DLL1 siRNAs (DLL1-si1/2), negative control siRNA (Ctr-si) or not-transfected (NT) as indicated. (A-B) At 55–70 hours after transfection, cells at 80–90% confluency were scratched, and wound closure was evaluated by microscopy at various time points. Representative images taken at the indicated times post-wounding from three independent experiments are shown. The graph represents mean percentage values (+ SD) of wound closure at each analyzed time point from scratches of these assays. (C) MDA-MB-231 cells were collected 72 hours after transfection and equal cell numbers were added to the upper chamber of 8-μm-pore membranes coated with matrigel and their invasion was measured. Representative fields of crystal-violet-stained cells that invaded to the lower surface of the membranes are shown in each condition. The graphs show mean percentage values (± SD) of invading cells of three independent experiments. *, *P* < 0.05, compared with Ctr-siRNA transfected cells.

We next performed scratch wound-healing assays to study the effect of DLL1 downregulation in BC cell motility. To do this, scratches were performed in monolayers of MCF-7 and MDA-MB-231 cells, non-transfected (NT) or transfected with DLL1 or control siRNAs 55 to 70 hours post-transfection and wound closure was followed by microscopy. Due to the fact that BT474 cells grow in compact, slowly growing multi-layered colonies that rarely become confluent, the scratch assay could not be performed in this cell line. Consistent with their higher migratory phenotype, our results showed that the wound closure of MDA-MB-231 cells occurred much faster than those of MCF-7 cells [19]. Importantly DLL1 downregulation resulted in a significant delay in wound closure in MCF-7 (70–80% reduction) and MDA-MB-231 (20% reduction) cells (Fig 2A and 2B). Twenty-four hours after wounding MCF-7 cells transfected with DLL1-siRNA1 presented 9% wound closure, when compared to approximately 50% of wound closure in Ctr-siRNA and NT cells, respectively. Seventy-two hours after wounding, MCF-7 cells transfected with DLL1-siRNA1 showed only 20% wound closure, when compared to more than 85% closure in control cells (transfected with Ctr-siRNA or NT) (Fig 2A). In MDA-MB-231 cells transfected with DLL1-siRNA1 and -siRNA2 only 50–55% wound closure occurred 20 hours after wounding (75 h after transfection) while control NT or transfected cells with Ctr-siRNA presented 63% and 74% wound closure, respectively (Fig 2B).

We next used a matrigel transmembrane invasion assay to study the effect of DLL1 downregulation in the invasive properties of MCF-7, BT474, and MDA-MB-231 cells. For this, non-transfected or cells transfected with DLL1-siRNAs and Ctr-siRNA were seeded in transwells, at different time points after transfection, and their invasion capability evaluated. In MDA-MB-231 cells, our results demonstrated that DLL1 downregulation by both DLL1 siRNAs (72 hours after transfection) decreased the number of invading MDA-MB-231 cells by an average of 25%, when compared to control cells transfected with Ctr-siRNA (Fig 2C). In agreement with published data [21] and due to the very low- or non-invasive phenotype of MCF-7 and BT474 cells in the absence of invasion-stimulating factors [19,22], no invading MCF-7 and BT474 cells were observed in these matrigel assays in all conditions tested (data not shown).

Altogether, these results suggest that DLL1 downregulation impairs BC cell migration and MDA-MB-231 invasive cellular properties.

### DLL1 downregulation impairs proliferation of luminal A MCF-7 BC cells

**Fig 3:**
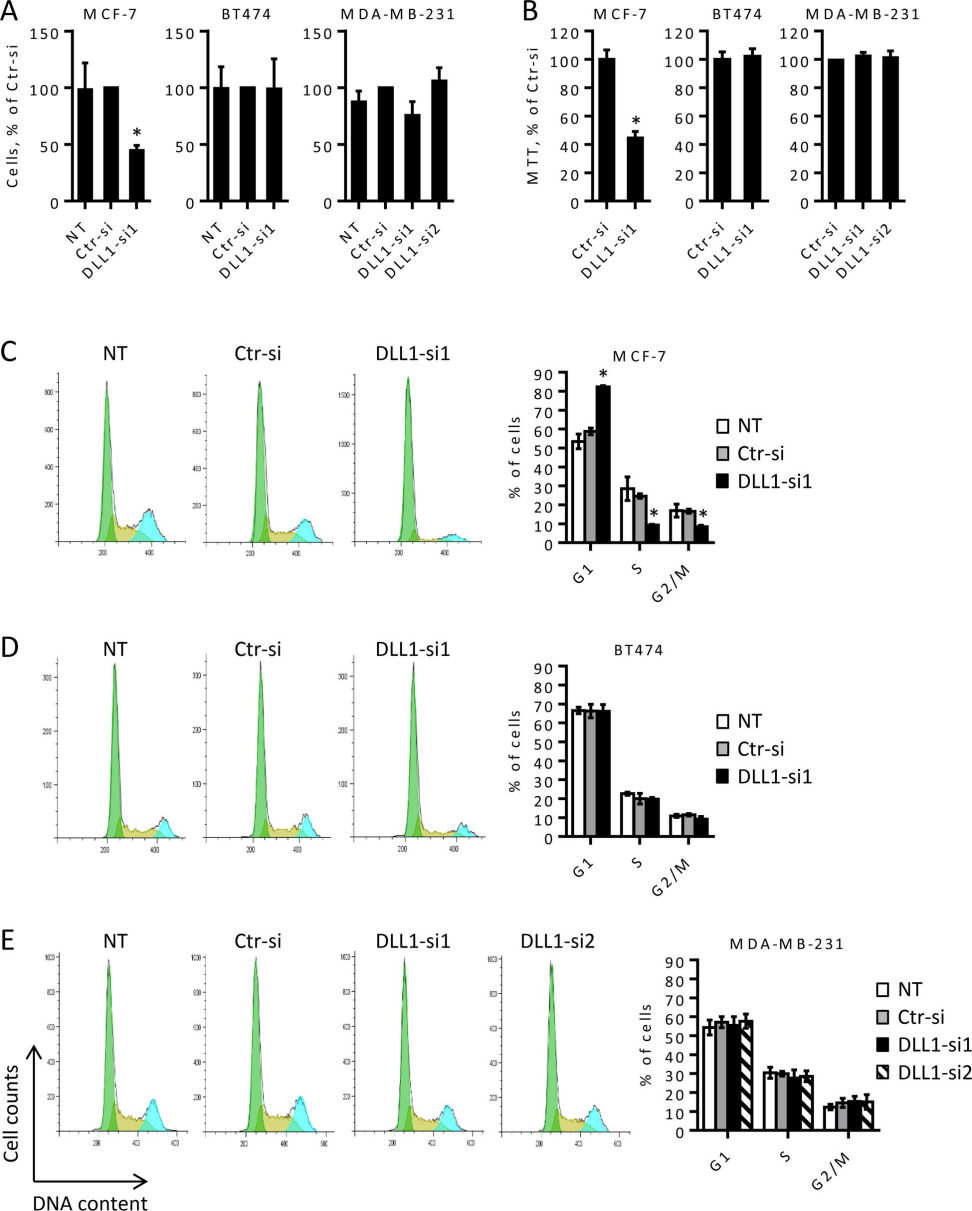
DLL1 downregulation in MCF-7 cells decreases proliferation and induces cell cycle arrest in the G1 phase. MCF-7, BT474, and MDA-MB-231 cells were not transfected (NT) or transfected with DLL1-siRNA (DLL1-si1/2), or negative control (Ctr) siRNA as indicated. (A-B) Cellular growth was determined by microscopy using the trypan blue exclusion (A) and the MTT (B) methods at 72 hours after transfection in MCF-7 and 72 up to120 hours in BT474 and MDA-MB-231 cells. Values were calculated as relative percentage of cells transfected with Ctr-siRNA, in each assay for each cell type. The graphs represent data (mean ± SD) from at least three independent assays. (C-E) At 86 hours post-transfection cells were collected, fixed, stained with propidium iodide, and DNA content was analyzed by flow cytometry. Representative histograms showing the effect of DLL1 downregulation in cell cycle progression from these assays are shown. Graph shows mean percentage (+ SD) of cells in each phase of the cell cycle of four independent assays. *, *P* < 0.05, compared with cells transfected with Ctr-siRNA.

DLL1 has been reported to be critical for proliferation in some cancer cells, such as glioma and melanoma cells [23,24]. Thus, we next evaluated the effect of DLL1 downregulation on MCF-7, BT474, and MDA-MB-231 cell growth. To do so, cells were transfected with DLL1-siRNA or Ctr-siRNA and cell growth was evaluated by trypan blue exclusion by microscopy and by the MTT assay at various time points starting 48 hours after transfection. Our results showed that DLL1 downregulation significantly decreased MCF-7 cell growth at 48 and 72 hours after transfection when compared to cells transfected with Ctr-siRNA. A higher reduction was detected at 72 hours, with a 50% decrease in cell growth observed in the MCF-7 cells (Fig 3A and 3B). Assessment of BT474 and MDA-MB-231 cell growth from 48 up to 120 hours after transfection showed that DLL1 downregulation did not significantly affect the proliferation of these cells (Fig 3A and 3B).

### DLL1 downregulation promotes G1 arrest and induces apoptosis of luminal A MCF-7 BC cells

The significant decrease in the proliferation of MCF-7 cells upon DLL1 downregulation (Fig 3A and 3B) suggested that DLL1 influences MCF-7 cell cycle progression. Thus, we next examined cell cycle profiles of BC cells after transfection with DLL1-siRNA or Ctr-siRNA by flow cytometry. Consistent with an impairment of proliferation with DLL1-siRNA1, analysis of cell cycle progression of MCF-7 cells performed at 72, 86 and 98 hours after transfections showed that DLL1 downregulation caused a cell arrest in the G1 phase and a decrease of cells in the S and G2/M phases (panel 1 in S1 Fig). At 86 hours after transfection, MCF-7 cells transfected with DLL1-siRNA1 showed a 40% increase in cell number in the G1 phase, and a 60% and 50% reduction in the S and G2/M phases of the cell cycle, respectively, when compared to cells transfected with Ctr-siRNA or non-transfected cells (Fig 3C). In agreement with a lack of an effect of DLL1 downregulation in BT474 and MDA-MB-231 cell proliferation (panels 2–3 from Fig 3A and 3B), cell cycle analysis of these cells demonstrated that DLL1 siRNA-mediated downregulation did not impact BT474 and MDA-MB-231 cell cycle progression (Fig 3D and 3E and panels 2–3 in S1 Fig). The number of cells in the different phases of the cell cycle were similar amongst cells transfected with the DLL1siRNAs, the Ctr-siRNA and the respective non-transfected cells.

**Fig 4:**
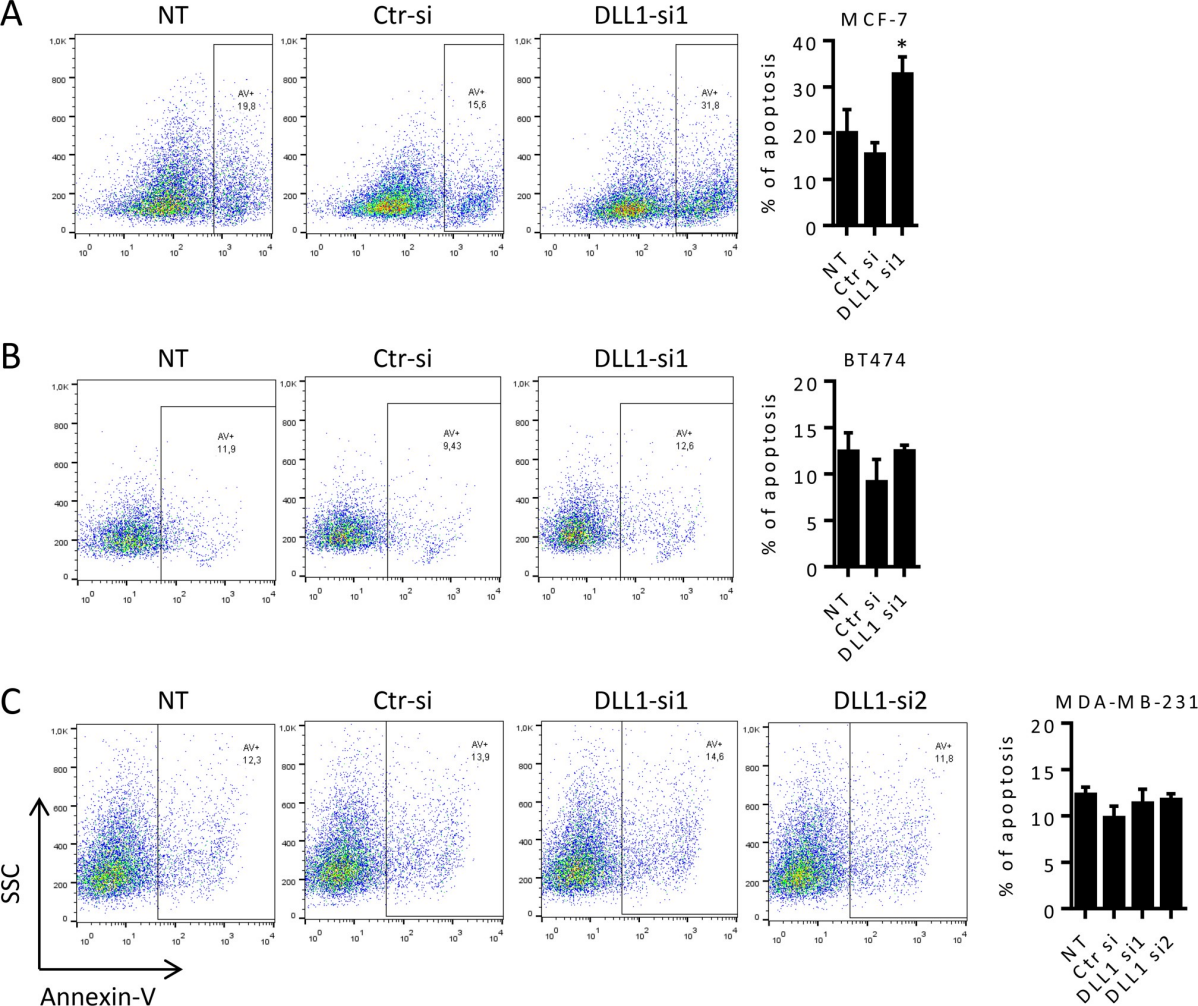
DLL1 downregulation promotes apoptosis of MCF-7 cells. (A-C) MCF-7, BT474 and MDA-MB-231 cells were not-transfected (NT) or transfected with DLL1-siRNAs (DLL1-si1/2), or negative control (Ctr) siRNA. At 92 hours (MCF-7) and 120 hours (BT474 and MDA-MB-231) after transfection cells were collected, stained with annexin-V and apoptosis analyzed by flow cytometry. Representative dot plots showing the percentage of apoptosis at each condition are represented. Graph shows percentage of apoptosis (mean + SD) of at least three independent assays, each one performed in triplicate. *, *P* < 0.05, compared with cells transfected with Ctr-siRNA.

Accumulation of cells in the sub-G1 population was observed in MCF-7 cells upon DLL1-siRNA1 transfection at later time points, when compared to cells transfected with Ctr-siRNA and NT cells, suggesting the induction of apoptosis. To confirm this, we then examined the effect of DLL1 downregulation in MCF-7 apoptosis. Cells were collected at 72, 92 and 120 hours post transfection, stained with annexin-V and analyzed by flow cytometry. Our data showed that MCF-7 cells transfected with DLL1-siRNA1 exhibited a 2-fold increase in apoptosis when compared to control cells transfected with Ctr-siRNA and NT cells at 92 hours (Fig 4A) and 120 hours (data not shown) after transfection. Assessment of apoptosis in BT474 and MDA-MB-231 cells up to 120 hours after transfection with DLL1-siRNAs, Ctr-siRNA and in NT cells showed that DLL1 downregulation did not induce apoptosis in these cells (Fig 4B and 4C).

Altogether, these data demonstrate that DLL1 has diverse effects in the human BC cell lines under study and suggests different mechanisms in the mode of action of DLL1.

### DLL1 downregulation in MCF-7 cells increases the expression of genes that promote cycle arrest and decreases those involved in the promotion of cell cycle progression and survival

**Fig 5:**
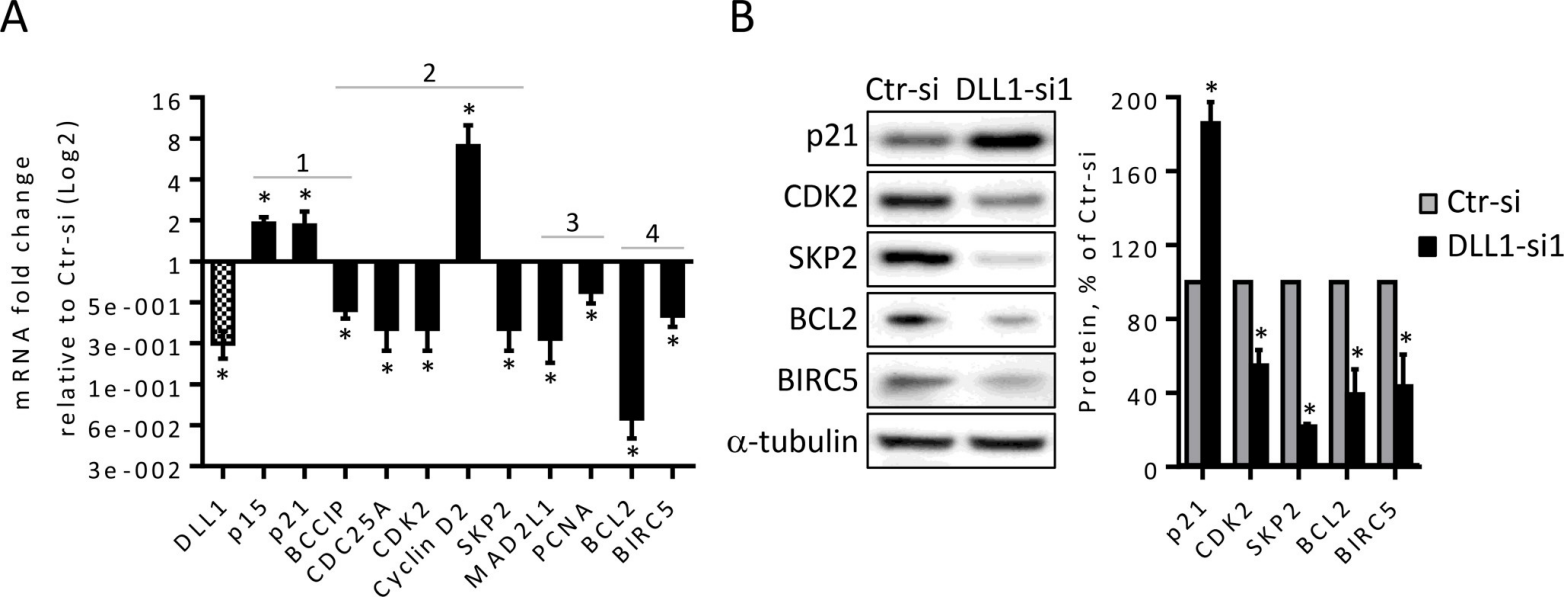
DLL1 downregulation in MCF-7 cells upregulate genes involved in cell cycle arrest and represses genes that promote cell cycle progression, proliferation and survival. Cells were transfected with DLL1-siRNA1 or Ctr-siRNA. (A) At 55–70 hours following transfection total RNA was extracted and the expression of genes that control cell cycle arrest (1), cell cycle transition (2), proliferation (3), and survival (4) were quantified by qRT-PCR. The values were normalized against the HPRT1 mRNA levels in the same sample and calculated as fold change relative to control cells transfected with Ctr-siRNA. Results are represented as mean values (+ SD) from four independent assays. (B) The expression levels of the indicated proteins were determined by immunoblotting 72 hours after transfection in total soluble protein extracts. α-tubulin was used as an internal control. The graph shows relative expression of these proteins in cells transfected with DLL1-siRNA1 as compared to Ctr-siRNA transfected cells of at least three independent experiments. *, *P* < 0.05, compared with cells transfected with Ctr-siRNA.

To explore the mechanisms through which DLL1 may exert its functional effects in MCF7 cells, we investigated the effect of siRNA-mediated DLL1 downregulation in the expression of genes that control the cell cycle. To this end, mRNA from MCF-7 cells transfected with DLL1-siRNA or control siRNA were isolated at 53 and 60 hours after transfection and the expression of 84 key genes involved in cell cycle control were quantified using a human cell cycle PCR array. We found that DLL1 downregulation in MCF7 cells is associated with a two-fold or greater change in 13 cell cycle genes. These findings were further confirmed by qRT-PCR in three independent assays at various time points starting at 53 h post-transfection. The effect of DLL1 downregulation on the expression of PCNA gene, associated with cell proliferation and shown to be highly expressed in almost all tumors, BC included [25], was also evaluated. The results showed that MCF-7 cells transfected with DLL1-siRNA1, which displayed a 75% decrease in DLL1 mRNA levels, presented significantly lower levels of several genes that promote cell cycle transition, proliferation and cell survival in comparison to cells transfected with Ctr-siRNA (Fig 5A). For instance, DLL1 downregulation increased by 2-fold the mRNA levels of genes involved in cell cycle arrest p21 and p15 [26], and decreased by 3-fold the expression of the G1/S cell cycle progression gene CDK2, whose activity is enhanced in BC and was shown to be involved in BC formation [26]. Moreover, DLL1 downregulation caused a 2-fold reduction in the expression levels of CDC25A and SKP2 genes, which are often overexpressed in breast tumors and involved in BC pathogenesis [27,28]. Interestingly, the expression levels of Cyclin D2, a gene involved in G1/S transition¸ with very low mRNA expression levels in MCF-7 cells (as shown in this study) and often intensely downregulated in BC [29], was significantly decreased (4-11-fold) upon DLL1 downregulation. The expression of BCCIP, a gene suggested to suppress tumor initiation but required for tumor progression [30], was reduced by 2-fold upon DLL1 downregulation. The mRNA levels of the proliferation-promoting genes PCNA and mostly MAD2L1, shown to be overexpressed in BC and associated with aggressive tumors and worse prognosis and BC cell proliferation [31], were also reduced by siRNA-mediated DLL1 downregulation (1.7- and 3.7-fold, respectively). The levels of BIRC5/Survivin, expressed in most human cancers, but absent in normal tissues, and shown to promote proliferation and inhibition of apoptosis of cancer cells including BC [32,33], were also significantly reduced by DLL1 downregulation (2-fold). The expression levels of BCL2, a gene that cooperates with pro-proliferative signal to support BC initiation and progression and therapeutic resistance [34], were dramatically decreased (14.6-fold) upon DLL1 downregulation. Furthermore, the effect of DLL1 downregulation in the protein levels of p21, CDK2, SKP2, BCL2 and BIRC5 was also evaluated by immunoblotting. Consistent with the mRNA levels, the protein levels of p21 showed a 2-fold increase upon DLL1 downregulation while those of CDK2, BCL2, BIRC5 and mostly SKP2 were significantly reduced (Fig 5B). Since the expression levels of cyclin D2 are extremely low in MCF-7 cells, this protein was undetectable by immunoblotting.

These results show that DLL1 downregulation modulates gene expression in MCF-7 cells in a manner consistent with the promotion of cell cycle arrest at the G1 phase and induction of apoptosis, and consequently suggests that DLL1 expression leads to the suppression of these processes.

### Treatment of MCF-7 cells with recombinant DLL1 increases their proliferation

**Fig 6:**
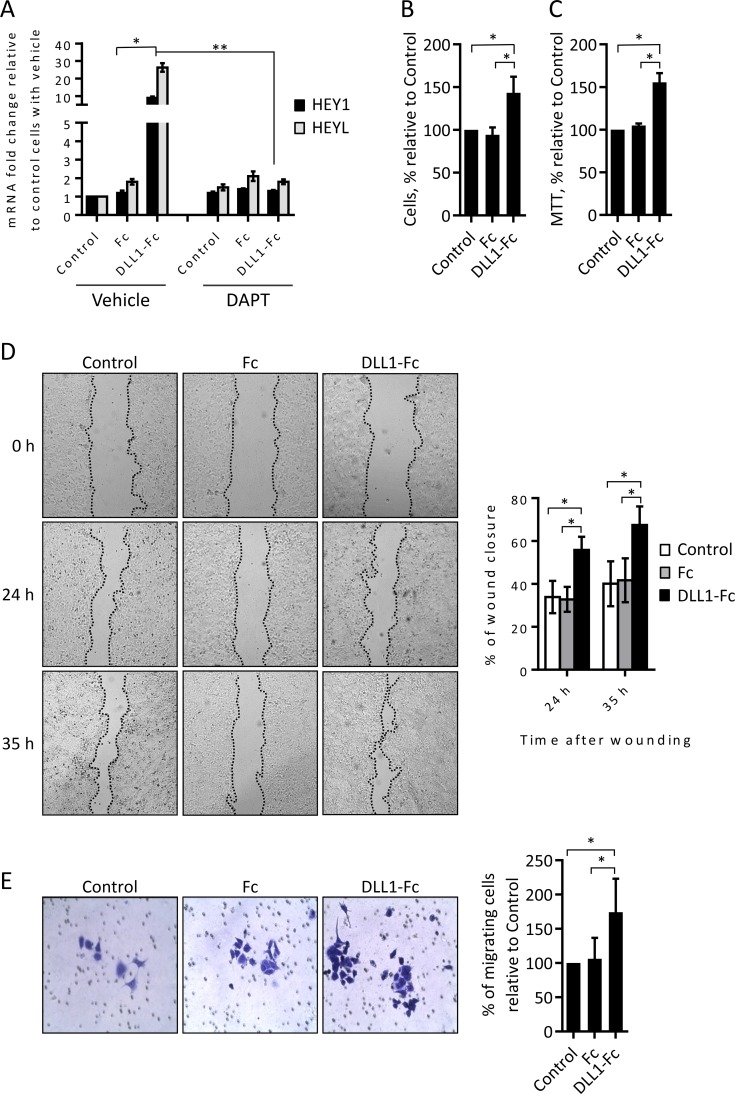
Human recombinant DLL1 activates the Notch pathway and promotes MCF-7 cell proliferation and migration. (A) MCF-7 cells were plated in tissue culture plates not coated (control) or pre-coated with DLL1-Fc or Fc proteins in the absence (vehicle) or the presence of the Notch pathway inhibitor DAPT. Total RNA was extracted 17 hours thereafter and the expression levels of the indicated Notch-target genes were quantified by qRT-PCR, and calculated as fold change relative to control non-treated cells. Graph represents data (mean + SD) from three independent experiments. *, *P* < 0.05, compared with cells treated with Fc control protein. **, *P* < 0.05, compared with cells treated with DLL1-Fc without DAPT. (B-C) At 48–72 hours following cell seeding as described above in the absence of DAPT, cellular growth was determined by trypan exclusion (B) and the MTT (C) methods. Values were calculated as percentage relative to those obtained in non-treated control cells. (D) Monolayers of control not-treated or cells treated with DLL1-Fc or Fc proteins for 72 hours were scratched and wound closure was measured at the indicated time points. Representative pictures taken at 0, 24 and 35 hours post-scratches are shown. The graph represents percentage values (mean + SD) of wound closure at each time point from scratches of three independent experiments. (E) Equal numbers of control non-treated or treated cells with DLL1-Fc or Fc proteins were added to the upper chamber wells with 8.0-μm pore membranes and allowed to migrate for 72 hours. Representative images of migrating cells are shown. The graph represents percentage values (mean + SD) of migrating cells of three independent experiments. *, *P* < 0.05, compared with controls not treated or with Fc protein. *P* values in (A) were calculated by using Student’s t test. *P* values shown in (B-E) were calculated using one-way ANOVA.

Overall, the results described above show that DLL1 downregulation has a higher impact in MCF-7 cells. Thus, we next validated the role of DLL1 in MCF-7 cells. Recombinant cell free immobilized ligands have been employed to stimulate the Notch pathway and elicit biological effects in various cells [35,36]. Accordingly, and in order to perform similar assays human recombinant DLL1-Fc protein, containing the DLL1 full extracellular domain fused to the Fc portion of human IgG1, was produced in mammalian cells. The Fc region of human IgG1 alone was also obtained to be used as a control protein. The ability of DLL1-Fc protein to activate the Notch signaling pathway in MCF-7 cells was evaluated by measuring the expression levels of the direct Notch-target genes HEY1 and HEYL [4] by qRT-PCR. DLL1-Fc protein was able to significantly increase the expression of HEY1 and HEYL, when compared to control cells not treated or treated with Fc protein (Fig 6A). Furthermore, pharmacological inhibition of Notch pathway activation by the treatment with the γ-secretase inhibitor DAPT (5 μM) suppressed the DLL1-Fc-mediated HEY1 and HEYL induction (Fig 6A), confirming that DLL1-Fc protein induced the expression of these genes through a mechanism which is dependent on the activation of Notch. The effect of DLL1-Fc protein in MCF-7 cell growth was analyzed by trypan blue exclusion and MTT assays. Cell count at various time points, starting 48 hours after cell incubation, showed a 50% increase in the number of MCF-7 cells incubated with DLL1-Fc when compared to control MCF-7 cells or cells incubated with Fc protein alone (Fig 6B). Similar results were obtained in the MTT assay (Fig 6C). Assessment of cell motility in the scratch wound-healing assays showed that a faster wound closure (2-fold increase) occurred in MCF-7 cells treated with DLL1-Fc protein than in control cells not treated or treated with the Fc protein (Fig 6D). Twenty-four hours after wounding, MCF-7 cells treated with DLL1-Fc showed an average of 55% wound closure when compared to 30% wound closure in control cells or cells treated with Fc protein alone. Thirty-five hours after wounding, the differences in the closure of the wounds were maintained, with MCF-7 cells incubated with DLL1-Fc showing 65% wound closure when compared to 40% in the control non-treated cells and in the cells incubated with Fc protein. Next, we confirmed the effect of DLL1-Fc in MCF-7 cell migration by performing transwell assays. As such, MCF-7 cells not treated or incubated with DLL1-Fc or Fc alone for 72 hours were seeded in transwells and their migration evaluated at various time points. Our results showed that incubation with DLL1-Fc increased the number of migrating MCF-7 cells, 72 hours after seeding, by an average of 75% when compared to the control non-treated cells or cells stimulated with the Fc protein (Fig 6E).

Overall, these results further demonstrate that DLL1 promotes MCF-7 cell proliferation and migration.

## Discussion

Accumulating evidence indicates that DLL1 might be involved in the development and progression of various types of cancer. For example, DLL1 expression is significantly higher in oligodendrogliomas than in normal brain cells and is associated with glioma cell proliferation and survival [23]. In childhood neuroblastomas with MYCN gene amplification, DLL1 is highly expressed and implicated in the onset of this type of cancer by promoting cell proliferation and maintaining their undifferentiated status [37]. In multiple myeloma, DLL1-mediated Notch activation has been shown to increase cell proliferation, promote clonogenic growth *in vitro*, accelerate tumor growth initiation [20], and contribute to drug resistance [38]. Other studies implicated DLL1 in choriocarcinoma tumor growth and invasion [39] and in melanoma tumor metastasis [24]. Based on these evidences, and on the observation that DLL1 expression is generally increased in human breast cancers when compared to normal breast tissues [8], we reasoned that DLL1 could play a role in the tumorigenic process of BC. In this study, we used three different *in vitro* models to analyze the effect of DLL1 in BC. We found that DLL1 is significantly more expressed in luminal A MCF-7 and B BT474 BC cells when compared to TNBC MDA-MB-231 cells, and that DLL1 downregulation decreases the carcinogenic features of these cells.

Functional assays revealed that DLL1 downregulation reduced the colony formation ability of luminal A MCF-7 and luminal B BT474 BC cells, but had no significant impact in the colony forming ability of MDA-MB-231 cells (Fig 1). These results suggest that DLL1 is required for the initial growth phases of these luminal BC cells. This hypothesis is consistent with data showing that DLL1-Notch pathway activation increased the number of colonies of multiple myeloma cells [20]. Cell motility studies performed in MCF-7 and MDA-MB-231 cells showed that DLL1 downregulation decreased migration of MCF-7 and MDA-MB-231 BC cells (Fig 2). In addition, DLL1 downregulation decreased the invasion potential of MDA-MB-231 cells (Fig 2). Interestingly, these results support the role of DLL1 in promoting migration and invasion of MDA-MB-231 cells, which are highly aggressive [19]. Indeed, according to reported data [16] DLL1 might promote MDA-MB-231 cell invasion and migration through mechanisms that rely on the expression of the metalloproteases MMP9 and MMP3, and VEGF. Moreover, DLL1 downregulation significantly decreased the proliferation of MCF-7 cells but not of BT474 and MDA-MB-231 cells (Fig 3). As opposed to DLL1 downregulation, treatment of MCF-7 with DLL1 protein, that specifically induced Notch pathway activation, lead to increased cell proliferation and migration (Fig 6). These findings clearly indicate that DLL1 is involved in the promotion of MCF-7 proliferation and migration. The results showing that DLL1 downregulation reduced BT474 colony forming ability but not their proliferation under normal culture conditions suggest that DLL1 is required for the initial growth of these cells and colony formation but not for proliferation. Overall, these results are consistent with reported data indicating that DLL1 promotes tumor growth of luminal BC but not TNBC cells [15].

The inhibition of MCF-7 cell proliferation by DLL1 downregulation was a consequence of cell cycle growth arrest, as evidenced by the increase in the number of cells in the G1 phase with the concomitant decrease of cells in the S and G2/M phases (Fig 3). Interestingly it has been shown that ERα receptor, highly expressed in luminal A BC but absent in TNBC, influences BC cell proliferation through the modulation of the S and G2/M phases of the cell cycle [40]. In addition, it has recently been proven that DLL1 exerts its function in BC cells through the ER receptor. Accordingly, the absence of ER expression in MDA-MB-231 cells and the low levels of this receptor in BT474, compared to MCF-7 cells [15], might underlie the fact that DLL1 downregulation did not impact MDA-MB-231 and BT474 cell cycle progression/proliferation. Cell cycle arrest of MCF-7 cells upon DLL1 downregulation was followed by augmented apoptosis (Fig 4), indicating an association of these events. Similar to other cancer types such as glioma and multiple myeloma [20,23], these findings suggest that DLL1 contributes to the carcinogenic features of MCF-7 cells by promoting cell proliferation and survival. In addition, and consistent with a lack of influence in cell proliferation, DLL1 downregulation had no impact in cell cycle progression and survival of BT474 and MDA-MB-231 cells (Figs 3 and 4). Taken together, these results reveal that DLL1 can have dissimilar effects in BC cells from different biological subtypes, and this is in agreement with published data [15]. The different cellular effects of DLL1 downregulation observed in MCF-7, BT474 and MDA-MB-231 cells are likely due to their genetic, epigenetic, and phenotypic variabilities and are in line with the cell context-dependent mode of action of the Notch signaling pathway [4,5].

Having identified a crucial role for DLL1 in decreasing MCF-7 cell proliferation and survival, we evaluated the effect of DLL1 downregulation in the transcription of major regulatory genes of these processes. Our findings revealed that DLL1 downregulation modulated the expression of cell cycle and survival genes (Fig 5) in a mode consistent with the promotion of cell cycle arrest, growth inhibition, and induction of apoptosis providing a mechanism, at least partially, for its mode of action in luminal A MCF-7 BC cells. DLL1 downregulation decreased the expression of CDC25A, CDK2, and SKP2 genes that are essential for G1/S cell cycle transition, PCNA and mostly MAD2L1 genes that promote proliferation, as well as the survival genes BCL2 and BIRC5. In addition, DLL1 downregulation increased the expression of p15 and p21, which block cell cycle progression at the G1/S transition, and Cyclin D2 (Fig 5). The MAD2L1 gene is highly expressed in BC and associated with poor prognosis and its downregulation has been shown to reduce BC cell proliferation [31]. PCNA is highly expressed in breast invasive ductal carcinoma [25], has been shown to be upregulated by the Notch signaling pathway, and to mediate increased proliferation of cells transfected with active Notch [41]. High expression levels of CDK2, SKP2 and CDC25A are detected in several types of cancer, BC included, and implicated in the promotion of cancer cell proliferation [27,28,42,43]. SKP2, one of the most downregulated genes, has been identified as a downstream target of the Notch signaling pathway and its upregulation by DLL4-Notch pathway activation is associated with the degradation of p21 and accelerated cell cycle progression [44]. p21 is repressed by Notch pathway activation [26,45] and DLL1-mediated Notch activation increased multiple myeloma cell proliferation by decreasing p21 [20]. Accordingly, repression of CDK2, CDC25A, SKP2 and induction of p21 likely contributed to MCF-7 cell cycle arrest at the G1 phase upon DLL1 downregulation. Interestingly, we observed that DLL1 downregulation considerably increased the mRNA levels of Cyclin D2, a G1/S transition gene, whose expression is very low or absent in some human cancers due to promoter hypermethylation [29,46]. The loss of Cyclin D2 expression is associated with BC evolution [29], and poor prognosis of prostate cancer [46]. Moreover, Cyclin D2 overexpression has been shown to inhibit cell cycle progression [47]. These findings demonstrated that cyclin D2 has tumor suppressive effects and suggest that its upregulation could also contribute, to some extent, for the herein observed effects in MCF-7 cells upon DLL1 downregulation.

BIRC5 (Survivin) is a cell survival gene regulated by the Notch signaling pathway [48], highly expressed in invasive and *in situ* ductal carcinomas, associated with poor prognosis, and shown to induce high proliferation of MCF-7 cells [32,33]. These observations may suggest that reduction of BIRC5 also contributed to cell cycle arrest and the induction of apoptosis in MCF-7 cells upon DLL1 downregulation. BCL2 is an anti-apoptotic protein essential for the normal development and homeostasis of the breast that is often overexpressed in various BC subtypes. In the context of ER^+^ BC patients, BCL2 upregulation has been shown to mediate tamoxifen resistance [34]. Interestingly, BCL2 has been shown to be a direct target of the Notch signaling pathway in a BC cell model [49]. The expression of this gene was highly decreased by the downregulation of DLL1, suggesting that this may accounted for the increased apoptosis observed in MCF-7 cells upon DLL1 downregulation.

Uncontrolled cellular proliferation and evasion of apoptosis are two of the hallmarks of cancer [50], shown to be amongst the mechanisms via which aberrant Notch signaling exerts its oncogenic potentials in many cancers, including BC [5]. Given that the Notch oncogenic function in human cancers is frequently mediated by upregulation of genes that support increased cell growth and survival, it is plausible that DLL1 promotes proliferation and survival of MCF-7 luminal A BC cells by mechanisms mediated partly through the modulation of the expression of the genes indicated above.

In conclusion, the results from our study suggest that DLL1 contributes to the pro-carcinogenic features of human BC MCF-7, BT474 and MDA-MB-231 cells. In MCF-7 cells, DLL1 supports proliferation and survival, partly through mechanisms that rely on the modulation of the expression of key genes controlling these cellular processes (e.g. p21, CDK2, SKP2, BCL2, and BIRC5). In BT474 cells, DLL1 promotes clonogenic growth and initial growth stages, and in MDA-MB-231 cells it potentiates migration and invasion. Our findings shed light on the role of DLL1 in different human BC cells and unravel possible mechanisms through which DLL1 exerts its function in MCF-7 cells.

## Supporting information

S1 Fig Effect of DLL1 downregulation on the cell cycle progression of MCF-7, BT474 and MDA-MB-231 cells. Cells were not transfected (NT), transfected with DLL1-siRNAs or negative control (Ctr) siRNA as indicated. At 72, 86 and 98 hours following transfections cells were collected, fixed, stained with propidium iodide, and DNA content was evaluated by flow cytometry. DLL1 downregulation in MCF-7 cells, but not in BT474 and MDA-MB-231 cells, resulted in an increase in the number of cells in the G1 phase population and a decrease in the number of cells in the S and G2/M phases populations. The graph show mean percentage of cells (± SD) in each phase of the cell cycle at each analyzed time point from triplicate samples in one of three independent experiments for each cell line.
(TIF)

## References

[1] Bettaieb A, Paul C, Plenchette S, Shan J, Chouchane L, et al (2017) Precision medicine in breast cancer: reality or utopia? J Transl Med 15: 139 10.1186/s12967-017-1239-z 28623955 PMC5474301

[2] Dai X, Li T, Bai Z, Yang Y, Liu X, et al (2015) Breast cancer intrinsic subtype classification, clinical use and future trends. Am J Cancer Res 5: 2929–2943. 26693050 PMC4656721

[3] Kopan R, Ilagan MX (2009) The canonical Notch signaling pathway: unfolding the activation mechanism. Cell 137: 216–233. 10.1016/j.cell.2009.03.045 19379690 PMC2827930

[4] Capaccione KM, Pine SR (2013) The Notch signaling pathway as a mediator of tumor survival. Carcinogenesis 34: 1420–1430. 10.1093/carcin/bgt127 23585460 PMC3697894

[5] Aster JC, Pear WS, Blacklow SC (2017) The Varied Roles of Notch in Cancer. Annu Rev Pathol 12: 245–275. 10.1146/annurev-pathol-052016-100127 27959635 PMC5933931

[6] Politi K, Feirt N, Kitajewski J (2004) Notch in mammary gland development and breast cancer. Semin Cancer Biol 14: 341–347. 10.1016/j.semcancer.2004.04.013 15288259

[7] Chakrabarti R, Celia-Terrassa T, Kumar S, Hang X, Wei Y, et al (2018) Notch ligand Dll1 mediates cross-talk between mammary stem cells and the macrophageal niche. Science 360. 10.1126/science.aan4153 PMC7881440 29773667

[8] Mittal S, Subramanyam D, Dey D, Kumar RV, Rangarajan A (2009) Cooperation of Notch and Ras/MAPK signaling pathways in human breast carcinogenesis. Mol Cancer 8: 128 10.1186/1476-4598-8-128 20030805 PMC2809056

[9] Hirose H, Ishii H, Mimori K, Ohta D, Ohkuma M, et al (2010) Notch pathway as candidate therapeutic target in Her2/Neu/ErbB2 receptor-negative breast tumors. Oncol Rep 23: 35–43. 19956862

[10] Reedijk M, Odorcic S, Chang L, Zhang H, Miller N, et al (2005) High-level coexpression of JAG1 and NOTCH1 is observed in human breast cancer and is associated with poor overall survival. Cancer Res 65: 8530–8537. 10.1158/0008-5472.CAN-05-1069 16166334

[11] Dickson BC, Mulligan AM, Zhang H, Lockwood G, O'Malley FP, et al (2007) High-level JAG1 mRNA and protein predict poor outcome in breast cancer. Mod Pathol 20: 685–693. 10.1038/modpathol.3800785 17507991

[12] Simoes BM, O'Brien CS, Eyre R, Silva A, Yu L, et al (2015) Anti-estrogen Resistance in Human Breast Tumors Is Driven by JAG1-NOTCH4-Dependent Cancer Stem Cell Activity. Cell Rep 12: 1968–1977. 10.1016/j.celrep.2015.08.050 26387946 PMC4594158

[13] Xing F, Okuda H, Watabe M, Kobayashi A, Pai SK, et al (2011) Hypoxia-induced Jagged2 promotes breast cancer metastasis and self-renewal of cancer stem-like cells. Oncogene 30: 4075–4086. 10.1038/onc.2011.122 21499308 PMC3145824

[14] Kontomanolis E, Panteliadou M, Giatromanolaki A, Pouliliou S, Efremidou E, et al (2014) Delta-like ligand 4 (DLL4) in the plasma and neoplastic tissues from breast cancer patients: correlation with metastasis. Med Oncol 31: 945 10.1007/s12032-014-0945-0 24696220

[15] Kumar S, Srivastav RK, Wilkes DW, Ross T, Kim S, et al (2018) Estrogen-dependent DLL1-mediated Notch signaling promotes luminal breast cancer. Oncogene. 10.1038/s41388-018-0562-z PMC6756232 30442981

[16] Shui Y, Yu X, Duan R, Bao Q, Wu J, et al (2017) miR-130b-3p inhibits cell invasion and migration by targeting the Notch ligand Delta-like 1 in breast carcinoma. Gene 609: 80–87. 10.1016/j.gene.2017.01.036 28163094

[17] Durocher Y, Perret S, Kamen A (2002) High-level and high-throughput recombinant protein production by transient transfection of suspension-growing human 293-EBNA1 cells. Nucleic Acids Res 30: E9 10.1093/nar/30.2.e9 11788735 PMC99848

[18] Silva G, Aboussekhra A (2015) p16(INK4A) inhibits the pro-metastatic potentials of osteosarcoma cells through targeting the ERK pathway and TGF-beta1. Mol Carcinog 55: 525–536. 10.1002/mc.22299 25728247

[19] Holliday DL, Speirs V (2011) Choosing the right cell line for breast cancer research. Breast Cancer Res 13: 215 10.1186/bcr2889 21884641 PMC3236329

[20] Xu D, Hu J, Xu S, De Bruyne E, Menu E, et al (2012) Dll1/Notch activation accelerates multiple myeloma disease development by promoting CD138+ MM-cell proliferation. Leukemia 26: 1402–1405. 10.1038/leu.2011.332 22094583

[21] Phannasil P, Thuwajit C, Warnnissorn M, Wallace JC, MacDonald MJ, et al (2015) Pyruvate Carboxylase Is Up-Regulated in Breast Cancer and Essential to Support Growth and Invasion of MDA-MB-231 Cells. PLoS One 10: e0129848 10.1371/journal.pone.0129848 26070193 PMC4467472

[22] Comsa S, Cimpean AM, Raica M (2015) The Story of MCF-7 Breast Cancer Cell Line: 40 years of Experience in Research. Anticancer Res 35: 3147–3154. 26026074

[23] Purow BW, Haque RM, Noel MW, Su Q, Burdick MJ, et al (2005) Expression of Notch-1 and its ligands, Delta-like-1 and Jagged-1, is critical for glioma cell survival and proliferation. Cancer Res 65: 2353–2363. 10.1158/0008-5472.CAN-04-1890 15781650

[24] Zhang JP, Li N, Bai WZ, Qiu XC, Ma BA, et al (2014) Notch ligand Delta-like 1 promotes the metastasis of melanoma by enhancing tumor adhesion. Braz J Med Biol Res 47: 299–306. 10.1590/1414-431X20143368 24714813 PMC4075293

[25] Qiu X, Mei J, Yin J, Wang H, Wang J, et al (2017) Correlation analysis between expression of PCNA, Ki-67 and COX-2 and X-ray features in mammography in breast cancer. Oncol Lett 14: 2912–2918. 10.3892/ol.2017.6516 28927045 PMC5588100

[26] Otto T, Sicinski P (2017) Cell cycle proteins as promising targets in cancer therapy. Nat Rev Cancer 17: 93–115. 10.1038/nrc.2016.138 28127048 PMC5345933

[27] Cangi MG, Cukor B, Soung P, Signoretti S, Moreira G Jr., et al (2000) Role of the Cdc25A phosphatase in human breast cancer. J Clin Invest 106: 753–761. 10.1172/JCI9174 10995786 PMC381390

[28] Wang Z, Fukushima H, Inuzuka H, Wan L, Liu P, et al (2012) Skp2 is a promising therapeutic target in breast cancer. Front Oncol 1. 10.3389/fonc.2011.00057 PMC3263529 22279619

[29] Evron E, Umbricht CB, Korz D, Raman V, Loeb DM, et al (2001) Loss of cyclin D2 expression in the majority of breast cancers is associated with promoter hypermethylation. Cancer Res 61: 2782–2787. 11289162

[30] Huang YY, Dai L, Gaines D, Droz-Rosario R, Lu H, et al (2013) BCCIP suppresses tumor initiation but is required for tumor progression. Cancer Res 73: 7122–7133. 10.1158/0008-5472.CAN-13-1766 24145349 PMC3918420

[31] Wang Z, Katsaros D, Shen Y, Fu Y, Canuto EM, et al (2015) Biological and Clinical Significance of MAD2L1 and BUB1, Genes Frequently Appearing in Expression Signatures for Breast Cancer Prognosis. PLoS One 10: e0136246 10.1371/journal.pone.0136246 26287798 PMC4546117

[32] Boidot R, Vegran F, Jacob D, Chevrier S, Gangneux N, et al (2008) The expression of BIRC5 is correlated with loss of specific chromosomal regions in breast carcinomas. Genes Chromosomes Cancer 47: 299–308. 10.1002/gcc.20533 18181175

[33] Hinnis AR, Luckett JC, Walker RA (2007) Survivin is an independent predictor of short-term survival in poor prognostic breast cancer patients. Br J Cancer 96: 639–645. 10.1038/sj.bjc.6603616 17285125 PMC2360044

[34] Williams MM, Cook RS (2015) Bcl-2 family proteins in breast development and cancer: could Mcl-1 targeting overcome therapeutic resistance? Oncotarget 6: 3519–3530. 10.18632/oncotarget.2792 25784482 PMC4414133

[35] Sahlgren C, Gustafsson MV, Jin S, Poellinger L, Lendahl U (2008) Notch signaling mediates hypoxia-induced tumor cell migration and invasion. Proc Natl Acad Sci U S A 105: 6392–6397. 10.1073/pnas.0802047105 18427106 PMC2359811

[36] Varnum-Finney B, Wu L, Yu M, Brashem-Stein C, Staats S, et al (2000) Immobilization of Notch ligand, Delta-1, is required for induction of notch signaling. J Cell Sci 113 Pt 23: 4313–4318. 11069775 10.1242/jcs.113.23.4313

[37] Bettinsoli P, Ferrari-Toninelli G, Bonini SA, Prandelli C, Memo M (2017) Notch ligand Delta-like 1 as a novel molecular target in childhood neuroblastoma. BMC Cancer 17: 352 10.1186/s12885-017-3340-3 28525978 PMC5438559

[38] Xu D, Hu J, De Bruyne E, Menu E, Schots R, et al (2012) Dll1/Notch activation contributes to bortezomib resistance by upregulating CYP1A1 in multiple myeloma. Biochem Biophys Res Commun 428: 518–524. 10.1016/j.bbrc.2012.10.071 23111325

[39] Pang RT, Leung CO, Lee CL, Lam KK, Ye TM, et al (2013) MicroRNA-34a is a tumor suppressor in choriocarcinoma via regulation of Delta-like1. BMC Cancer 13: 25 10.1186/1471-2407-13-25 23327670 PMC3561246

[40] JavanMoghadam S, Weihua Z, Hunt KK, Keyomarsi K (2016) Estrogen receptor alpha is cell cycle-regulated and regulates the cell cycle in a ligand-dependent fashion. Cell Cycle 15: 1579–1590. 10.1080/15384101.2016.1166327 27049344 PMC4934046

[41] Wang N, Liu W, Tan T, Dong CQ, Lin DY, et al (2017) Notch signaling negatively regulates BMP9-induced osteogenic differentiation of mesenchymal progenitor cells by inhibiting JunB expression. Oncotarget 8: 109661–109674. 10.18632/oncotarget.22763 29312637 PMC5752550

[42] Liu W, Wu M, Du H, Shi X, Zhang T, et al (2018) SIRT6 inhibits colorectal cancer stem cell proliferation by targeting CDC25A. Oncol Lett 15: 5368–5374. 10.3892/ol.2018.7989 29552180 PMC5840749

[43] Ronchini C, Capobianco AJ (2001) Induction of cyclin D1 transcription and CDK2 activity by Notch(ic): implication for cell cycle disruption in transformation by Notch(ic). Mol Cell Biol 21: 5925–5934. 10.1128/MCB.21.17.5925-5934.2001 11486031 PMC87311

[44] Sarmento LM, Huang H, Limon A, Gordon W, Fernandes J, et al (2005) Notch1 modulates timing of G1-S progression by inducing SKP2 transcription and p27 Kip1 degradation. J Exp Med 202: 157–168. 10.1084/jem.20050559 15998794 PMC2212905

[45] Noseda M, Chang L, McLean G, Grim JE, Clurman BE, et al (2004) Notch activation induces endothelial cell cycle arrest and participates in contact inhibition: role of p21Cip1 repression. Mol Cell Biol 24: 8813–8822. 10.1128/MCB.24.20.8813-8822.2004 15456857 PMC517869

[46] Padar A, Sathyanarayana UG, Suzuki M, Maruyama R, Hsieh JT, et al (2003) Inactivation of cyclin D2 gene in prostate cancers by aberrant promoter methylation. Clin Cancer Res 9: 4730–4734. 14581343

[47] Meyyappan M, Wong H, Hull C, Riabowol KT (1998) Increased expression of cyclin D2 during multiple states of growth arrest in primary and established cells. Mol Cell Biol 18: 3163–3172. 10.1128/mcb.18.6.3163 9584157 PMC108898

[48] Knight BB, Oprea-Ilies GM, Nagalingam A, Yang L, Cohen C, et al (2011) Survivin upregulation, dependent on leptin-EGFR-Notch1 axis, is essential for leptin-induced migration of breast carcinoma cells. Endocr Relat Cancer 18: 413–428. 10.1530/ERC-11-0075 21555376 PMC3361735

[49] Ferreira AC, Suriano G, Mendes N, Gomes B, Wen X, et al (2012) E-cadherin impairment increases cell survival through Notch-dependent upregulation of Bcl-2. Hum Mol Genet 21: 334–343. 10.1093/hmg/ddr469 21989054

[50] Hanahan D, Weinberg RA (2011) Hallmarks of cancer: the next generation. Cell 144: 646–674. 10.1016/j.cell.2011.02.013 21376230

