# Supplementary figures and images for: The Notch ligand DLL1 exerts carcinogenic features in human breast cancer cells
Source: PLoS One. 2019 May 20;14(5):e0217002. doi: 10.1371/journal.pone.0217002 (PMC6527237; doi:10.1371/journal.pone.0217002)

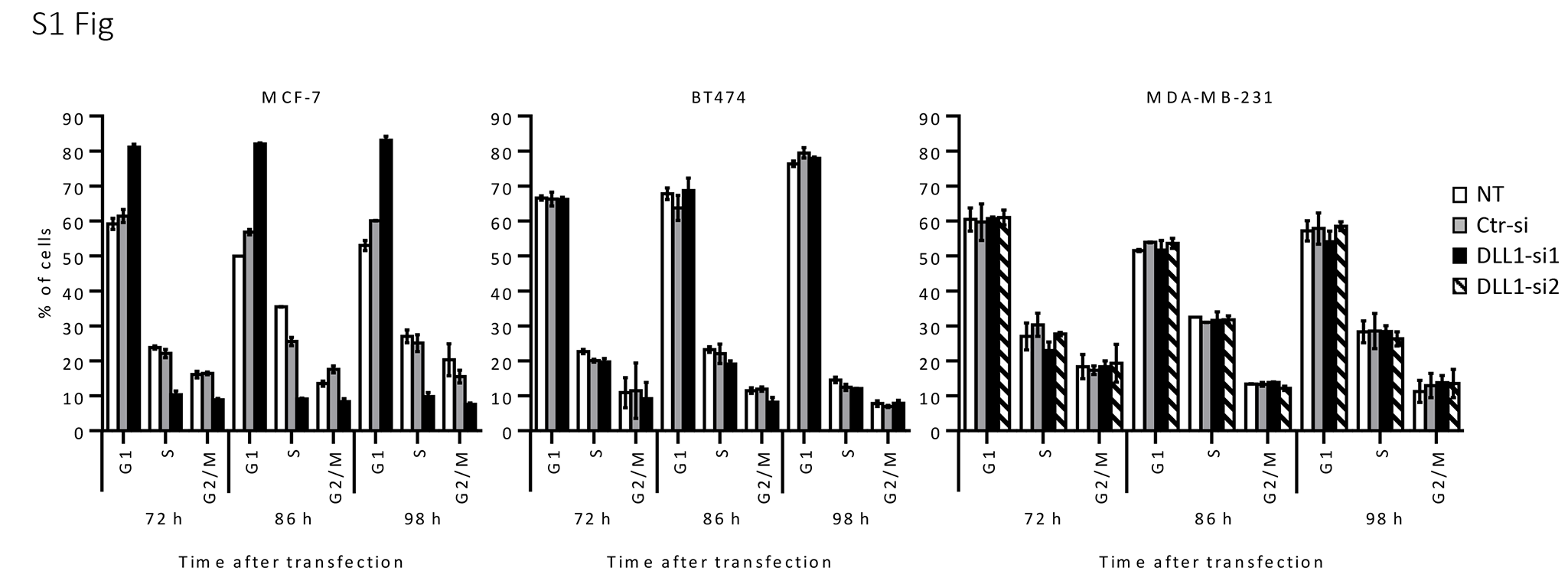

Supplement: S1 Fig — Cells were not transfected (NT), transfected with DLL1-siRNAs or negative control (Ctr) siRNA as indicated. At 72, 86 and 98 hours following transfections cells were collected, fixed, stained with propidium iodide, and DNA content was evaluated by flow cytometry. DLL1 downregulation in MCF-7 cells, but not in BT474 and MDA-MB-231 cells, resulted in an increase in the number of cells in the G1 phase population and a decrease in the number of cells in the S and G2/M phases populations. The graph show mean percentage of cells (± SD) in each phase of the cell cycle at each analyzed time point from triplicate samples in one of three independent experiments for each cell line. (TIF) [file pone.0217002.s001.tif]
